# Preparation and performance of composite agent with anti-seepage and friction reducing for pipe jacking in water-rich sand stratum

**DOI:** 10.1371/journal.pone.0341338

**Published:** 2026-02-13

**Authors:** Hairong Gu, Kai Wu, Peng Zhang, Jiejun Yuan, Xiao Zhang, Kui Shi

**Affiliations:** 1 State Grid Jiangsu Electric Power Co., Ltd., Nanjing Jiangsu, China; 2 School of Transportation Engineering, Nanjing Tech University, Nanjing, Jiangsu, China; 3 Xuzhou Power Supply Branch of State Grid Jiangsu Electric Power Co., Ltd., Xuzhou, Jiangsu, China; Jazan University College of Engineering, SAUDI ARABIA

## Abstract

Pipe jacking in water-rich sandy strata is frequently suffered by rapidly increasing seepage-induced hazards (e.g., groundwater inrush at working shafts) in jacking lubrication, which is because their limited anti-seepage capacity of conventional bentonite-based lubricants. In order to enhance the lubrication and anti-seepage performance of lubricants along the pipe–soil annulus, this study developed a composite agent with anti-seepage and friction reducing, where graphite(SM) is used as lubrication, Na_2_CO_3_ is used as dispersibility, polyacrylamide(PAM) is used as thixotropy, polyisocyanates and polyols are used as primary agents, triethanolamine(TEA) and dibutyltin dilaurate (DBTDL) are used as catalysts, and sodium silicate is used as water resistance due to the gelation effect at lower temperatures. The preparation method of the composite anti-seepage and friction reducing agent was investigated, and its macro- and micro-scale performance tests were conducted. Additionally, the optimal mix ratio was determined using the range analysis method and entropy weight method. The research indicates that the optimized composition of the composite anti-seepage and friction reducing agent is as follows: SM(0.8%−1.0%), PAM (2.0%−2.5%), Na_2_CO_3_ (0.3%−0.5%), DBTDL (1.4%−1.6%), TEA(1.0%−1.5%), and Na_2_O·3.1SiO_2_ (15%−20%). Compared to traditional bentonite-based friction reducing agent, the funnel viscosity range is reduced by 55%, the filtration loss range is reduced by 25%, the water separation rate is decreased by 4.5% (approaching 0), the friction coefficient is lowered by 20% ~ 28%, and the permeability coefficient reaches 3.5 × 10^-^⁵-5.0 × 10^-^⁵ cm/s. This paper addresses the issues of high frictional resistance and difficult waterproofing in jacking construction through water-rich sand stratum. The proposed preparation process of composite agent provides an essential material of practical route for reducing jacking forces and eliminating water inrush risks during pipe jacking in water-rich sandy strata.

## 1. Introduction

Pipe jacking has become a mainstream trenchless method for installing municipal pipelines and utility tunnels in dense urban areas because it minimises surface disruption and environmental impacts while enabling long-distance drives. However, as long distance pipe jacking in water-rich sandy strata, construction risks shift from purely mechanical issues to coupled mechanical–hydraulic problems, including rapidly increasing jacking forces, loss of jacking face stability, and groundwater migration along the pipe–soil interface that can trigger inrush events at working shafts reception pits and pipe joint joint.

In engineering practice, friction reduction is primarily achieved by injecting thixotropic slurry into the annular gap between the outer wall of the pipeline and the soil body to convert dry friction to lubricated (wet) friction, improve over-cut support, and limit ground loss. Existing studies have focused on bentonite-based and polymer-modified slurry systems and their mix design, including variations in bentonite type, polymer additives and dispersants, and environmentally friendly modifiers to improve viscosity stability, reduce filtration loss and bleeding, and enhance friction-reduction efficiency [1–6]. As the primary component of friction-reducing agents, bentonite has been modified with additives such as Na_2_CO_3_, PAM, hydroxymethyl cellulose, and xanthan gum [7–12]. Cui, et al. [13] addressed bentonite’s environmental impact by developing biodegradable pre-gelatinized starch (PGS)-modified reducers, analyzing PGS content effects. Liu et al.[14] incorporated 2% nano-silica into traditional reducers to enhance properties. Dai et al. [15] proposed water-soluble polymers (e.g., polyvinyl alcohol, partially hydrolyzed PAM) with inorganic crosslinkers to achieve dense film formation and flocculation. Yang et al. [16] developed a reducer for expansive soils comprising plant gum, potassium humate, CMC, and graphite powder to mitigate pipe wrapping caused by water absorption. Recent work continues to refine slurry design for challenging conditions (e.g., long-distance and large-diameter drives), including optimization of pipe jacking mud and evaluation of thixotropic additives such as hydroxyethyl cellulose [17–19].

Polyurethane (PU) grouts are widely used for rapid plugging because of their fast reaction and strong expansion sealing, and PU–water glass systems have been explored to improve water plugging efficiency and bonding performance. Recent advances in synthesis and modification (e.g., optimized viscosity, injectability) have expanded its application to fine-fracture grouting and low-permeability strata [20–23]. However, high reaction temperatures degrade PU’s anti-seepage properties. Zhang, Guan, Mei and Wang et al. [24–27] incorporated Na_2_O·3.1SiO_2_ to absorb reaction heat, reducing peak temperatures by 30%. Xue [28] optimized water-soluble (WPU) and oil-soluble PU (OPU) ratios, showing that blending polyols with varying molecular weights and functionalities enhances WPU microstructure and OPU crosslinking density. Li [29] developed PU magnetic capsules for dam piping control, combining hydrophobic/hydrophilic PU in hydroxypropyl methylcellulose (HPMC) shells with magnetic materials for delayed reaction and adsorption. Eco-friendly diluents (e.g., ethyl acetate) were introduced to reduce toxicity.

Despite the above progress, existing anti-seepage grouting materials are typically applied independently from lubrication slurries, and incompatibility between systems (e.g., phase separation, inadequate retention in the annulus, and mismatched setting time) can reduce effectiveness during continuous jacking. Therefore, an integrated material–process solution that addresses both friction reduction and seepage migration is still lacking. The motivate of this study is to proposes a composite anti-seepage and friction-reducing agent and an associated staged grouting process for pipe jacking in water-rich sand strata. The main contributions are: (i) development of a thixotropic friction reducer (SM–PAM–Na_2_CO_3_) compatible with a fast-setting PU–water glass anti-seepage system; (ii) a construction method using an inflatable sealing ring to improve annulus isolation and grout retention; (iii) a laboratory-based performance evaluation framework combining macro-indices (viscosity, filtration loss, bleeding, interface friction coefficient and permeability reduction) with microstructural observations; and (iv) formulation optimisation using orthogonal experiments, range analysis and entropy-weighted multi-criteria scoring to obtain recommended dosage ranges. These results provide a practical basis for simultaneously controlling jacking forces and mitigating water inrush risks in water-rich sandy strata.

## 2. Raw materials for preparation

### 2.1. Test sand

**Fig 1:**
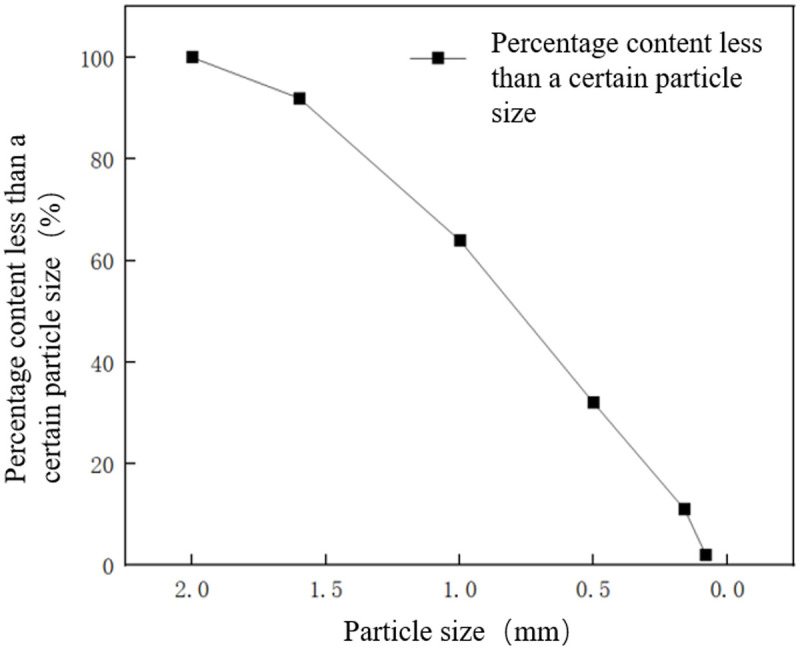
Particle gradation of sand.

Fig 1 shows the grading of test sand used in the test. The test sand has a density of 1.9 g/cm^3^, a water content of 24%, a porosity of 39%, and a particle size range of 0.075 to 2.0 mm. Its shear strength parameters are an internal friction angle of 32° and a cohesion of 1.2 kPa, with a permeability coefficient of 3 × 10^−4^ cm/s.

#### 2.2 Friction Reducer.

**Fig 2:**
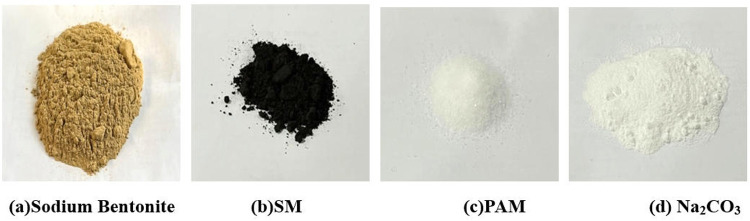
Raw materials for friction reducing agents. (a) Sodium Bentonite. (b) SM. (c) PAM. (d) Na2CO3.

The friction-reducing agent consists of SM, PAM, and Na_2_CO_3_, as illustrated in Fig 2.

Graphite is an opaque, extremely soft carbon-based mineral with a greasy and slippery texture. Its color ranges from iron-black to steel-gray, exhibiting various layered structural forms including crystalline, flaky, scaly, and striated configurations. The interlayer forces in graphite are governed by weak van der Waals interactions, which, combined with its layered structure, account for its excellent lubricating properties.

PAM is a polymer synthesized from acrylamide monomers. It can be classified into four types: non-ionic (NPAM), cationic (CPAM), anionic (APAM), and amphoteric (Amphoteric PAM). The anionic polyacrylamide, with the chemical formula (C_3_H_5_NO)n, features long molecular chains capable of forming bridges between particles. Its backbone contains numerous amide groups that can establish strong hydrogen bonds with various compounds, making it a highly effective flocculant.

Na_2_CO_3_ is a white, odorless inorganic powder. When dissolved in water, it ionizes or hydrolyzes to produce OH- ions. The dissociation yields carbonate ions (CO₃^2^⁻) and sodium ions (Na⁺), which at high concentrations can impede water molecule mobility and reduce the solution’s hydration capacity.

### 2.3. Anti-seepage agent

**Fig 3:**
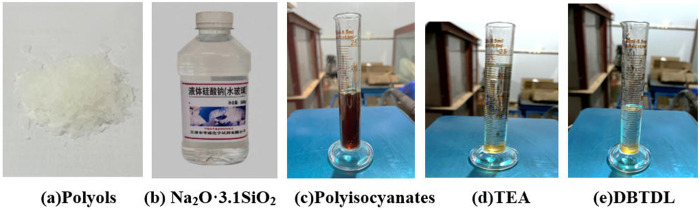
Raw materials of anti-seepage agent. (a) Polyols. (b) Na2O·3.1SiO2. (c) Polyisocyanates. (d) TEA. (e) DBTDL.

Polyurethane is primarily synthesized through chemical reactions between polyisocyanates and polyols. The engineering properties of polyurethane vary significantly depending on the raw materials and their proportions. The synthesis involves primary agents and additives. The primary agents include isocyanates and polyols: isocyanates provide reactive (-NCO) groups, while polyols supply hydroxyl (-OH) groups that react with isocyanates to form polyurethane segments. Additives encompass chain extenders, crosslinkers, catalysts, plasticizers, and curing agents. Chain extenders and crosslinkers promote linear chain growth and branching/crosslinking between molecular chains. Organotin catalysts accelerate the reaction between isocyanates and hydroxyl groups, whereas tertiary amine catalysts facilitate the reaction between isocyanates and water. The raw materials are shown in Fig 3.

#### 2.3.1. Primary agents.

The primary agents for polyurethane synthesis consist of polyisocyanates and polyols. Polyisocyanates (TDI, MDI, HDI) provide highly reactive isocyanate groups (-NCO), while polyols (polyether polyols, polyester polyols) offer hydroxyl (-OH) groups as reactive sites. These components undergo addition polymerization to form urethane linkages (-NH-COO-), constituting the backbone of the polyurethane structure.

Isocyanates (CHNO) are typically brownish liquids at room temperature. Common isocyanates used in polyurethane synthesis include toluene diisocyanate (TDI), diphenylmethane diisocyanate (MDI), hexamethylene diisocyanate (HDI), isophorone diisocyanate (IPDI), and polymethylene polyphenyl isocyanate (PAPI). The core functional group (-NCO) exhibits high reactivity and can react with hydroxyl groups in hydroxyl compounds, water, amines, and other active hydrogen-containing compounds to form urethanes, ureas, biurets, etc.

Polyether polyols are colorless, oily liquids whose molecular backbones consist of alkoxy groups connected by ether bonds (-O-), with terminal or side chains containing multiple hydroxyl (-OH) groups. Polymer polyols used in polyurethane synthesis mainly include polyether polyols and polyester polyols. Polyester polyols yield polyester-based polyurethanes with high adhesive strength, but their inherent poor hydrolysis resistance makes them unsuitable for waterproof materials. In contrast, polyether polyols, containing ether bonds in their molecular chains, exhibit superior hydrolysis resistance and are thus more appropriate for waterproof material synthesis.

#### 2.3.2. Additives.

Dibutyltin dilaurate (C_32_H_64_O_4_Sn, abbreviated as DBTDL) is an organotin catalyst that strongly promotes the NCO-OH reaction. It appears as a pale yellow, transparent, flammable liquid with a relative density of 1.066, freezing point of 8°C, density of 1.0425 kg/L, and flash point of 226.7°C. While insoluble in water, DBTDL dissolves readily in various organic solvents. Its structure comprises two dibutyltin groups (C₄H₉) and two laurate ester groups (OOC-C₁₁H₂₃).

Triethanolamine (C6H15NO3, abbreviated as TEA) is an organic amine catalyst containing three ethanol groups (-CH₂CH₂OH) and one amino group (-NH₂). This weakly alkaline, colorless oily liquid (pKa ≈ 7.82) exhibits properties of both tertiary amines and alcohols. At low temperatures, it reacts with organic acids to form salts, while at high temperatures it produces TEA esters. TEA can react with isocyanates and become incorporated into the cured molecular structure, leaving minimal free TEA after the reaction.

Sodium silicate (Na_2_O·3.1SiO_2_) is a water-soluble, transparent or translucent glassy solid formed by varying ratios of alkali metal oxides (Na₂O or K₂O) and SiO₂. It exists as sodium silicate (Na₂O·nSiO₂) or potassium silicate (K₂O·nSiO₂), where n represents the molar ratio of SiO₂ to alkali metal oxide, known as the silicate modulus. When used as a grouting material, Na_2_O·3.1SiO_2_ solution forms a gel under the catalytic action of curing agents. This experiment employed Na_2_O·3.1SiO_2_ with a modulus of 3.1.

## 3. Preparation and implementation process

### 3.1. Preparation process

According to the preparation protocol, precise quantities of bentonite, SM, PAM, Na_2_CO_3_, and purified water were weighed. The preparation was conducted in a temperature-controlled environment (20 ± 1°C) using a high-speed mixer at 1,500 rpm for 30 minutes to ensure homogeneous and stable dispersion of all raw materials in water, yielding a stable friction reducer.

For the anti-seepage grout preparation, the primary agents—polyisocyanate and polyether polyol—were thoroughly mixed first. Following the protocol, additives such as TEA and DBTDL were incorporated. The mixture was stirred at 600 rpm for 1.5–2 hours within a temperature range of 40–50°C to produce a polyurethane prepolymer. Finally, the prepolymer was blended with Na_2_O·3.1SiO_2_ and a measured amount of water to formulate the final anti-seepage grout.

### 3.2. Implementation process

To address the issue of friction reducer loss in conventional practices, this study proposes an integrated approach combining inflatable sealing ring technology and gradient-pressure grouting during pipe jacking. This method enables real-time grout replenishment through multiple injection ports per pipe segment and dynamic adjustment of the friction reducer-to-anti-seepage agent ratio in the composite material.

**Fig 4:**
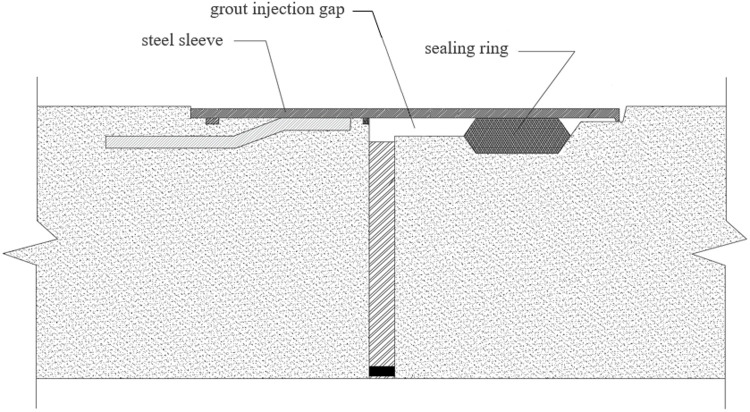
Schematic diagram of the structure of the joint part of the pipe section.

The inflatable sealing ring technology involves installing chloroprene rubber sealing rings at pipe joint connections. An installation gap naturally exists at the spigot-and-socket interface between pipe segments. Within this gap, a steel sleeve and sealing ring are positioned. The sealing ring’s deflated outer diameter is designed to be less than or equal to the pipe wall’s outer diameter, while its maximum compression displacement when inflated does not exceed half of its expanded outer diameter (as illustrated in Fig 4). When inflated, the sealing ring maintains a contact pressure exceeding 0.3 MPa. Its elastic deformation lifts the socket-end steel sleeve, creating an effective supporting structure that forms a grout injection gap between the pipe wall and steel sleeve. Grout is then injected into this gap. The inflated rubber sealing ring effectively seals the joint interface, confining the injected anti-seepage and friction-reducing agent and enabling the gradient-pressure grouting process. Finally, when the sealing ring is deflated, it contracts to tightly adhere to the pipe wall. This dual-action ensures: (1) complete filling of the gap with grout, and (2) prevention of grout leakage during subsequent pipe jacking operations.

Two-Stage Grouting Process:

Stage 1: After inflating the sealing rings at the jacking head segment, anti-seepage agent is injected to fill the gaps. The rings are then deflated to apply a gradient pressure ≤0.3 MPa, forcing the agent into the soil at the pipe-soil interface.

Stage 2: The rings are reinflated to inject the friction reducer, followed by pipe jacking. Only friction reducer replenishment is required for trailing segments to sustain friction reduction.

## 4. Performance test

### 4.1. Performance requirements

The performance indicators of conventional friction reducers primarily include Marsh funnel viscosity, filtration loss, and water separation rate. The Marsh funnel viscosity ensures good fluidity during transportation and grouting processes; filtration loss controls water infiltration into the formation, preventing dehydration-induced failure of the friction reducer; while water separation rate guarantees the formation of a stable gel structure after standing. Detailed performance requirements are specified in Table 1.

**Table 1:** Main performance requirements of composite anti-seepage and friction-reducing agent.

**Friction-reducing performance**	**Index**	**Requirements**	**Anti-seepage** **performance**	**Index**	**Requirements**
	Funnel viscosity	≥30s		Density (g/cm^3^)	≥1.05
	Filtration loss	≤25 ml/30 min		Viscosity (MPa·s)	≤1.0 × 10^3^
	Water separation rate	no water-separation after 24h standing		gel Time(s)	≤150
	Friction coefficient	0.35 ~ 0.4		Permeability coefficient	<10^–6 cm^/s

The polyurethane anti-seepage agent must meet the performance requirements and criteria listed in Table 1. The permeability coefficient of the polyurethane-standard sand mixture should be less than 10^−6^ cm/s to ensure effective blocking of groundwater seepage and adaptability to complex formation conditions. The three main performance indicators for polyurethane anti-seepage agents are: density, viscosity, and gel time.

### 4.2. Test plan

#### 4.2.1. Friction reduction performance test.

The sodium bentonite-based slurry was used as the control group for orthogonal experiments. Based on empirical data of sodium bentonite friction reducers, the typical concentration range is 2%−10%. Five gradient concentrations (2%, 4%, 6%, 8%, and 10%) were prepared according to the mass ratio of solid components to water to identify the optimal sodium bentonite concentration for friction reduction.

The orthogonal experimental design selected SM, PAM, and Na_2_CO_3_ as the three primary agents, each with five concentration levels. A standard L₂₅ orthogonal array was adopted. Key performance indicators including funnel viscosity, filtration loss, water separation rate, and friction coefficient, were measured to analyze the influence of material ratios on friction reduction performance. The detailed test matrix is provided in Table 2.

**Table 2:** Factor levels table for orthogonal test of friction reduction performance.

Factor levels	Factors		
	A.SM	B.PAM	C. Na_2_CO_3_
**1**	0.4%	1.0%	0.2%
**2**	0.6%	1.5%	0.3%
**3**	0.8%	2.0%	0.4%
**4**	1.0%	2.5%	0.5%
**5**	1.2%	3.0%	0.6%

#### 4.2.2. Anti-seepage performance test.

The orthogonal experiment fixed the equal-mass ratio of polyisocyanate and polyether polyol as the main components, while varying three additives: TEA, DBTDL, and Na_2_O·3.1SiO_2_. Each additive was tested at four concentration levels. The dosages of the amine-based and tin-based catalysts were designed as mass percentages of the polyol, whereas Na_2_O·3.1SiO_2_’s dosage was based on the polyurethane solution mass. An L₁₆ orthogonal array was employed. Performance metrics such as density, viscosity, gel time, and permeability coefficient were evaluated to determine the impact of material ratios on anti-permeability performance. The test matrix is outlined in Table 3.

**Table 3:** Factor levels table for orthogonal test of anti-permeability performance.

Factor levels	Factors		
	A.TEA	B.DBTDL	C.Na_2_O·3.1SiO_2_
**1**	0.3%	1.4%	5%
**2**	0.6%	1.6%	10%
**3**	0.9%	1.8%	15%
**4**	1.2%	2.0%	20%

### 4.3. Testing methods

#### 4.3.1. Funnel viscosity.

**Fig 5:**
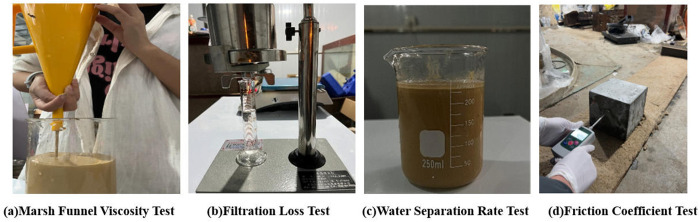
Test methods of friction-reducing performance. (a) Marsh Funnel Viscosity Test. (b) Filtration Loss Test. (c) Water Separation Rate Test. (d) Friction Coefficient Test.

Funnel viscosity primarily reflects the fluidity and supportability of the slurry. If the funnel viscosity of the friction reducer is too low, the slurry may provide insufficient support at the pipe-soil interface. Conversely, if the funnel viscosity is too high, the fluidity of the friction reducer decreases, leading to increased frictional resistance. A Marsh funnel viscometer was used, as shown in Fig 5(a). The procedure involved pouring 946 mL of slurry through a sieve into the funnel while blocking the bottom hole with a finger until the friction reducer leveled with the sieve. The bottom hole was then released, and a stopwatch was started. The time taken for the friction reducer in the beaker to reach the 964 mL mark was recorded in seconds (s).

#### 4.3.2. Filtration loss.

Filtration Loss reflects the degree of water leakage from the slurry into the formation under pressure. A reasonable filtration loss value ensures minimal water loss to maintain lubrication performance while effectively sealing soil pores to prevent formation instability. An API filter press was used, as shown in Fig 5(b). The tested friction reducer was poured into the cup until the liquid surface aligned with the marked line. A sealing ring and filter paper were placed sequentially, followed by the lid with a filter screen. The cup base was aligned with the slot on the cup body, rotated 90° to secure it, and the tightening screw was fastened. A 25 mL measuring cylinder was placed under the outlet to collect the filtrate. A pressure of over 1 MPa was applied using a hand pump, and the working pressure was maintained at 0.69 MPa by adjusting the pressure valve. After 30 minutes, the filtrate volume in the cylinder was recorded as the API filtration loss, measured in mL/30 min.

#### 4.3.3. Water separation rate.

The water-separation rate determines the stability of the friction reducer. A high water-separation rate may cause stratification, leading to sedimentation of solid particles and potential pipe blockage during grouting. A beaker was used to measure the water-separation rate, as shown in Fig 5(c). The tested friction reducer was poured into a 250 mL beaker up to a marked line and left undisturbed at room temperature for 24 hours. The volume change was observed, and the water-separation rate was calculated as the ratio of the volume change to the original volume. The required water-separation rate was 0.

#### 4.3.4. Friction coefficient.

The friction coefficient (μ) was used as the friction reduction index. A C50 concrete test block simulated the concrete of a jacking pipe, while standard sand simulated sandy soil. The concrete block measured 150 mm × 150 mm × 150 mm and weighed 7.816 kg, with the applied normal pressure simulating the vertical load on pipe sections during construction. Standard sand was evenly spread on a wooden board at a thickness of 50–60 mm and leveled repeatedly to ensure a flat and uniformly compacted surface. A digital push-pull gauge was used to move the concrete block at a constant speed, and the sliding friction force (F) was recorded. For grouting simulation, the prepared reagent was evenly spread on the sand layer, and the thickness of the slurry along the path was recorded. The field test setup is shown in Fig 5(d).

#### 4.3.5. SEM microscopic imaging.

A Hitachi S-4800 scanning electron microscope was used to observe the microstructure of the samples at magnifications of 5 μm, 10 μm, and 50 μm. The analysis focused on the polymer structures composed of SM, PAM, and Na_2_CO_3_, as well as the microstructural characteristics of polymers formed by isocyanate, polyol, TEA, DBTDL, and Na_2_O·3.1SiO_2_.

#### 4.3.6. Density and viscosity.

**Fig 6:**
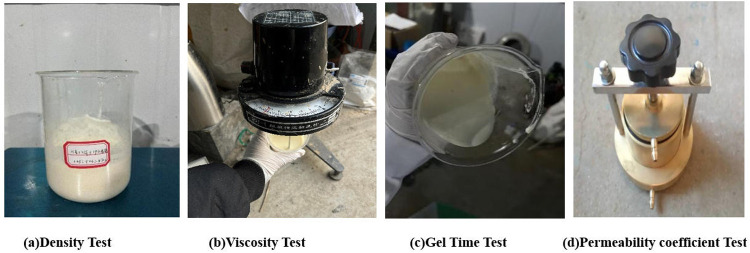
Test methods of anti-seepage performance. (a) Density Test. (b) Viscosity Test. (c) Gel Time Test. (d) Permeability coefficient Test.

The density of polyurethane was tested according to GB/T 6343−2009, as shown in Fig 6(a), to determine the density of the main agent. Viscosity was measured using an NDJ-I rotary viscometer following GB/T 2794−1995, as shown in Fig 6(b).

#### 4.3.7. Gel time.

The inverted cup method was used to measure the gel time of the slurry, following JC/T 2041−2010, as shown in Fig 6(c). Ten grams of isocyanate (Component A) and ten grams of polyol (Component B, mixed with catalysts, Na_2_O·3.1SiO_2_, and water according to orthogonal test ratios) were separately prepared. After thorough stirring, Components A and B were combined, and the stopwatch was started immediately upon complete mixing. The mixture was repeatedly poured between cups until it ceased flowing, and the elapsed time was recorded as the gel time.

#### 4.3.8. Permeability coefficient.

The permeability coefficients of standard sand and polyurethane–sand mixtures were tested using a TST-55 permeameter under variable head conditions, as shown in Fig 6(d). The procedure included: weighing 205 g of standard sand, preparing 10 g of polyol and 10 g of polyisocyanate as Component A, and other reagents as Component B per orthogonal test design. Components A and B were mixed uniformly with the sand to form the sample, which was then placed in the permeameter. The permeability coefficient was measured under a variable head ranging from 90 cm to 80 cm.

## 5. Experimental results

### 5.1. Control group

Three parallel experiments were conducted on five bentonite-based friction reducers with different concentrations to analyze the influence of sodium bentonite concentration on friction reduction performance, serving as a benchmark for orthogonal tests of composite friction reducers.

#### 5.1.1. Funnel viscosity.

**Fig 7:**
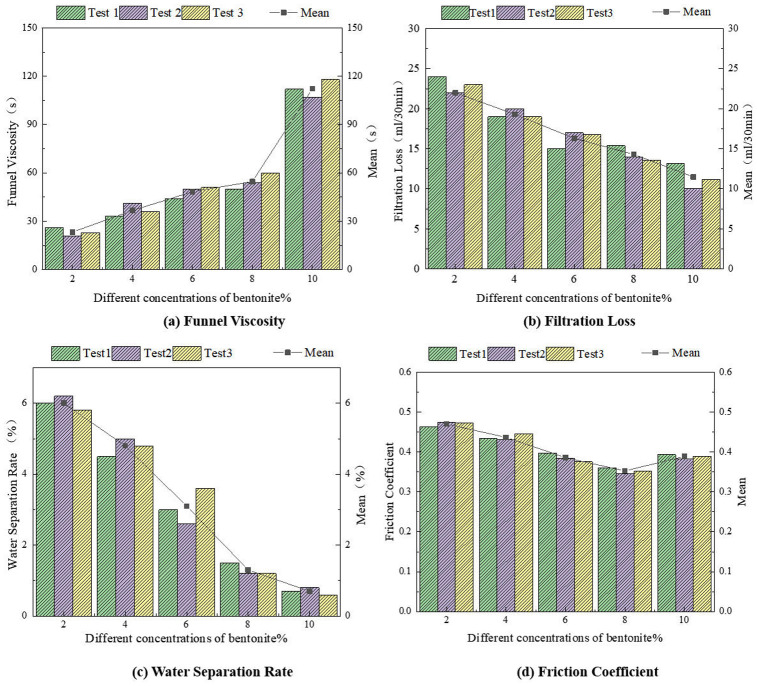
Relationship between performance and concentration of bentonite-based friction reducing agent. (a) Funnel Viscosity. (b) Filtration Loss. (c) Water Separation Rate. (d) Friction Coefficient.

As shown in Fig 7(a), the funnel viscosity of bentonite exhibited a nonlinear increasing trend as its concentration rose from 2% to 10%. Within the 2% ~ 8% concentration range, the viscosity increased gradually, with a growth rate of 19% per concentration gradient. However, when the concentration reached 8% ~ 10%, the funnel viscosity surged rapidly, resulting in poor fluidity of the friction reducer. The viscosity growth rate in this range reached 51% per concentration gradient.

#### 5.1.2. Filtration loss.

Fig 7(b) indicates that filtration loss decreased linearly with increasing bentonite concentration. At 2% concentration, the filtration loss was relatively high (22 mL/30 min). When the concentration reached 10%, the filtration loss decreased by 11.5 mL/30 min, with a growth rate of 12%–20% per concentration gradient.

#### 5.1.3. Water separation rate.

As illustrated in Fig 7(c), the water-separation rate followed a trend similar to filtration loss, showing an overall decline as the bentonite concentration increased from 2% to 10%. When the concentration reached 8%−10%, the water-separation rate approached zero, demonstrating the enhanced water-adsorption capacity of bentonite at higher concentrations.

#### 5.1.4. Friction coefficient.

Fig 7(d) reveals that the friction coefficient initially decreased and then slightly increased as the bentonite concentration rose from 2% to 10%. The interfacial friction coefficient between standard sand and the pipe was 0.5. At bentonite concentrations of 2%–6%, the friction coefficient ranged from 0.47 to 0.38, representing a reduction of 6%−24%. The lowest friction coefficient (0.353, a 29% reduction) was achieved at 8% concentration. However, when the concentration further increased to 10%, the friction coefficient slightly rebounded. This is attributed to the sharp rise in funnel viscosity beyond 8% concentration, which impaired fluidity and led to particle aggregation, ultimately reducing lubrication effectiveness and increasing friction.

### 5.2. Performance of friction reducer

Based on orthogonal test data, range analysis was employed to investigate the influence of raw material formulations (SM, PAM, Na₂CO₃) on friction reduction performance indicators.

#### 5.2.1. Funnel viscosity.

**Fig 8:**
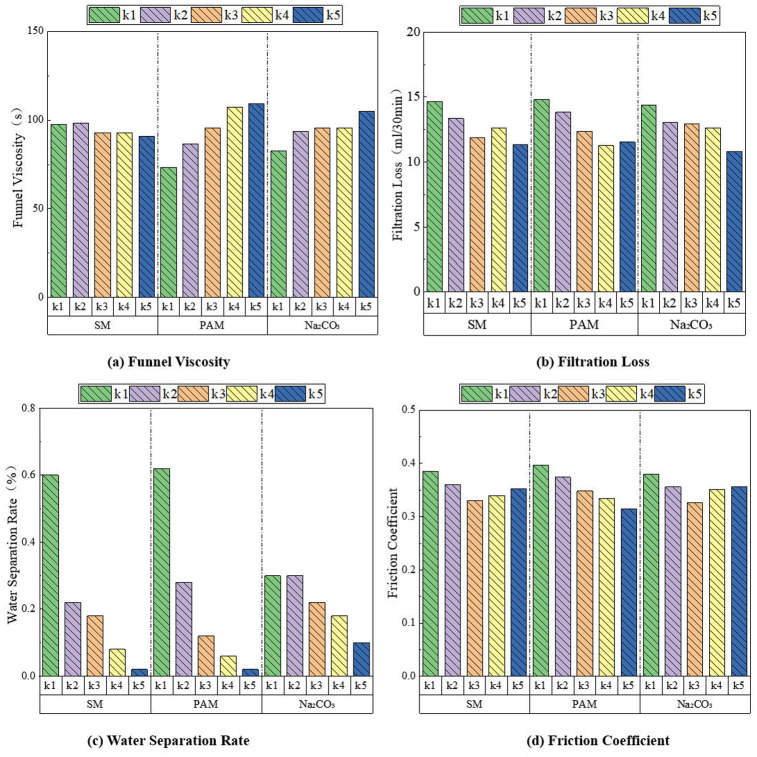
Influence of raw material ratio on the performance of friction reduction. (a) Funnel Viscosity. (b) Filtration Loss. (c) Water Separation Rate. (d) Friction Coefficient.

The order of influence of raw material composition on funnel viscosity was: PAM > SM > Na₂CO₃, as shown in Fig 8(a). The funnel viscosity exhibited a significant increasing trend with increasing PAM content. For SM additions, the viscosity generally increased, though the rate of increase slowed when SM content reached 0.6%−1.0%. With Na₂CO₃ additions, viscosity initially increased at concentrations below 0.3%, but showed a decreasing trend when Na₂CO₃ content exceeded 0.3%. The long-chain structure and polar amide groups of PAM contribute to thickening, water retention, anti-seepage and rheological modification in the composite friction reducer. When dissolved in water, PAM forms a dense network structure that increases particle movement resistance, thereby elevating funnel viscosity. SM particles function through physical filling and dispersion effects. Their lamellar structure restricts polymer chain segment movement, increasing viscosity. At SM contents of 0.6%−1.0%, localized ordered structures or weak flocculation may form, resulting in slower viscosity increase rates. Na₂CO₃ influences viscosity through pH regulation. Below 0.3% concentration, increased pH promotes PAM dissolution and dispersion, enhancing internal friction and indirectly raising viscosity. However, when Na₂CO₃ exceeds 0.3%, excessive ionic concentration disrupts PAM molecular chain entanglement, reducing internal friction resistance and consequently decreasing viscosity.

#### 5.2.2. Filtration loss.

The order of influence of raw material composition on filtration loss was: Na₂CO₃ > PAM > SM, as shown in Fig 8(b). When the Na₂CO₃ content was 0.3%−0.5%, the filtration loss showed a decreasing trend. When the PAM content was 1.0%−2.5%, the filtration loss decreased with increasing PAM content; when the PAM content was 2.5%−3.0%, the filtration loss increased with increasing PAM content. When the SM content was 0.4%−0.8%, the filtration loss decreased with increasing SM content; when the SM content was 0.8%−1.0%, the filtration loss increased with increasing SM content; when the SM content was 1.0%−1.2%, the filtration loss decreased with increasing SM content. Na₂CO₃ regulates through the synergistic effect of ionic strength and pH. Na⁺ promotes particle aggregation by compressing the electric double layer. When the ionic strength exceeds the critical value, the repulsive force between particles is completely shielded, achieving permeation equilibrium. The active groups such as amide groups on PAM molecular chains can adsorb on the surface of graphite particles and others. After multiple particles are adsorbed by polyacrylamide molecular chains, the bridging effect of molecular chains forms larger flocs, which fill the pores and reduce filtration loss. However, when PAM exceeds 2.5%, excessive PAM makes the friction reducer too viscous, which instead leads to an increase in filtration loss. SM mainly reduces filtration loss through physical filling of pore structures. Nevertheless, when SM reaches a certain content, the interaction between particles may cause agglomeration, which instead increases filtration loss.

#### 5.2.3. Water separation rate.

The influence order of raw material composition on water separation rate was: PAM > SM > Na₂CO₃, as shown in Fig 8(c). With increasing contents of SM, PAM and Na₂CO₃, the water separation rate exhibited a continuous decreasing trend. When PAM content exceeded 2.0% and SM content exceeded 0.6%, the decreasing rate of water separation slowed down. The increased PAM content could lock free water through hydrogen bonding and capillary action, significantly improving the funnel viscosity and water retention capacity of the mixture, thereby reducing water separation rate. However, when PAM exceeded 2.0%, excessive PAM molecular chains reached saturated adsorption on particle surfaces, leaving free chains unable to effectively participate in bridging, thus slowing down the decreasing trend of water separation rate. SM inhibited water migration through its lamellar structure, making it more difficult for water to quickly escape from the mixture, consequently reducing water separation rate. When SM content exceeded 0.6%, excessive SM content caused particle agglomeration, destroying suspension uniformity and resulting in a slower decrease of water separation rate. At initial low concentrations (0.1%−0.2%), Na₂CO₃ was insufficient to significantly alter the chemical environment, showing negligible effect on water separation rate. When Na₂CO₃ content exceeded 0.2%, excessive Na₂CO₃ indirectly destabilized the mixture through electrochemical regulation, slowing down the decreasing trend of water separation rate.

#### 5.2.4. Friction coefficient (*μ*).

The influence order of raw material composition on *μ* was: PAM > SM > Na₂CO₃, as shown in Fig 8(d). With increasing content of SM and Na₂CO₃, μ exhibited a trend of first decreasing and then increasing. In contrast, μ showed a continuous decreasing trend with increasing PAM content. The friction coefficient was reduced by approximately 4%−48%, with a maximum reduction of 52%. PAM molecular chains can form a lubricating adsorption film on material surfaces. As PAM content increases, the thickness and integrity of this adsorption film continuously improve, significantly reducing frictional resistance. SM possesses a layered structure with extremely low interlayer friction coefficients. At low concentrations (0.4%−0.8%), SM can uniformly disperse in the mixture to form a lubricating layer, thereby reducing the friction coefficient. However, at higher concentrations (0.8%−1.2%), stacking may occur between SM layers, increasing surface inhomogeneity and consequently raising the friction coefficient. Na₂CO₃ reduces the friction coefficient by adjusting pH to improve surface properties. At concentrations of 0.2%−0.4%, Na₂CO₃ optimizes surface lubricity and lowers the friction coefficient. However, excessive Na₂CO₃ content may lead to formation of uneven surface deposits, thereby increasing the friction coefficient.

### 5.3. Anti-seepage agent performance test

Based on orthogonal test data, range analysis method was used to analyze the influence of raw material ratio on anti-seepage performance indicators.

#### 5.3.1. Density.

**Fig 9:**
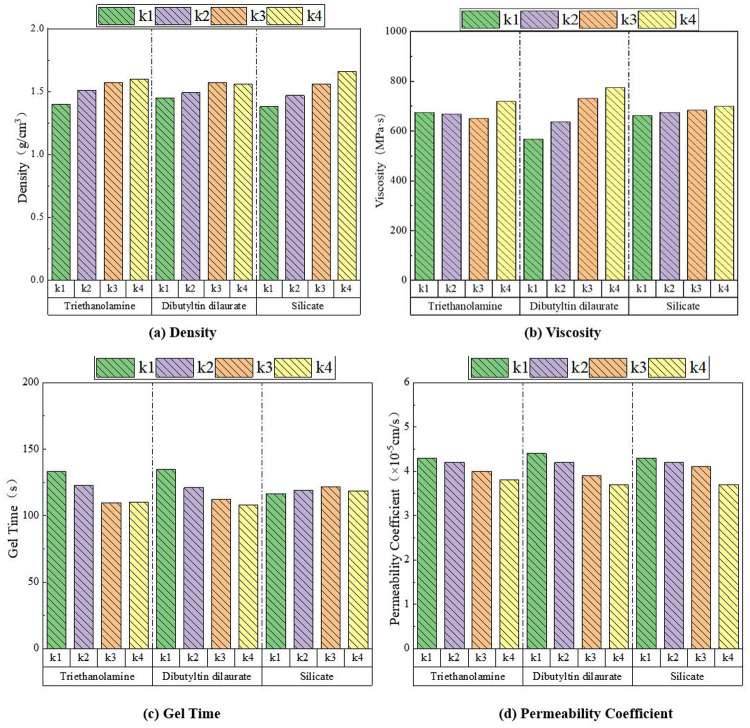
Influence of raw material ratio on anti-seepage performance. (a) Density. (b) Viscosity. (c) Gel Time. (d) Permeability Coefficient.

The influence degree of raw material ratio on density was: Na_2_O·3.1SiO_2_ > TEA > DBTDL, as shown in Fig 9(a). With the increase of TEA and Na_2_O·3.1SiO_2_ content, the density of the anti-seepage agent showed an overall upward trend. When the concentration of DBTDL was 1.4%−1.8%, the density first showed an upward trend, and when its concentration was 1.8%−2.0%, the density showed a slight downward trend. As an inorganic silicate, Na_2_O·3.1SiO_2_ has a significantly higher density than other organic components, directly affecting the density. TEA increases the pH value, accelerates the hydrolysis of Na_2_O·3.1SiO_2_ and the release of silicate ions, indirectly promotes the formation of Si-O-C bonds, and enhances the crosslinking density. DBTDL, as an organotin catalyst, accelerates the crosslinking reaction between -NCO and -OH groups in polyurethane and enhances the crosslinking density of molecular chains. However, its effect on improving density is limited, so DBTDL has the least influence on density.

#### 5.3.2. Viscosity.

The influence order of raw material composition on viscosity was: DBTDL > TEA > Na_2_O·3.1SiO_2_, as shown in Fig 9(b). With increasing content of DBTDL, the viscosity showed significant improvement. When TEA concentration was 0.3%−0.9%, the viscosity first exhibited a decreasing trend, while at 0.9%−1.2% concentration, the viscosity showed an increasing trend. With increasing Na_2_O·3.1SiO_2_ content, the viscosity demonstrated a slow rising trend. The enhanced interactions between molecular chains of DBTDL led to substantial viscosity increase. Meanwhile, DBTDL could accelerate the reaction between TEA and Na_2_O·3.1SiO_2_, promoting molecular chain crosslinking and polymerization, thereby causing rapid viscosity rise. TEA mainly reduced viscosity through catalytic reactions and salt formation. It could promote the dissolution and reactivity of Na_2_O·3.1SiO_2_. The reaction between TEA and Na_2_O·3.1SiO_2_ generated silicate gel, resulting in viscosity increase. The reactivity of Na_2_O·3.1SiO_2_ was regulated by pH value and catalysts, providing silicate ions to participate in gel-forming reactions, but its influence on viscosity was relatively limited.

#### 5.3.3. Gel time.

The influence degree of raw material composition on gel time was: DBTDL > TEA > Na_2_O·3.1SiO_2_, as shown in Fig 9(c). With the increase of DBTDL and TEA content, the gel time showed a decreasing trend; when the Na_2_O·3.1SiO_2_ concentration was 5%−15%, the gel time showed an increasing trend, and when the Na_2_O·3.1SiO_2_ concentration was 15%−20%, the gel time showed a decreasing trend. DBTDL can significantly accelerate the reaction between isocyanate (NCO) and polyols or water, thereby shortening the gel time. TEA mainly accelerates the reaction between NCO and water, shortening the gel time. The concentration and modulus of Na_2_O·3.1SiO_2_ also have certain effects on gel time, but are relatively weaker compared to DBTDL and TEA.

#### 5.3.4. Permeability coefficient.

The permeability coefficient measured for standard sand in the experiment was 5.35 × 10^−3^ cm/s. The permeability coefficient of the mixed soil composed of composite anti-seepage agent and standard sand ranged from 3.5 to 5.0 × 10^−5^ cm/s. The influence degree of raw material composition on permeability coefficient was: DBTDL > Na_2_O·3.1SiO_2_ > TEA, as shown in Fig 9(d). With the increase of DBTDL, Na_2_O·3.1SiO_2_ and TEA content, the permeability coefficient showed a decreasing trend. DBTDL has high catalytic efficiency and can rapidly form dense polyurethane network structures, effectively reducing porosity and connectivity, thus demonstrating excellent anti-seepage performance. Na_2_O·3.1SiO_2_ mainly plays filling and reinforcing roles. In addition, Na_2_O·3.1SiO_2_ can indirectly affect reaction rate and pore structure by adjusting pH value and ion concentration. The influence of TEA on permeability coefficient is relatively small.

### 5.4. SEM microscopic experiments

#### 5.4.1. Control group.

**Fig 10:**
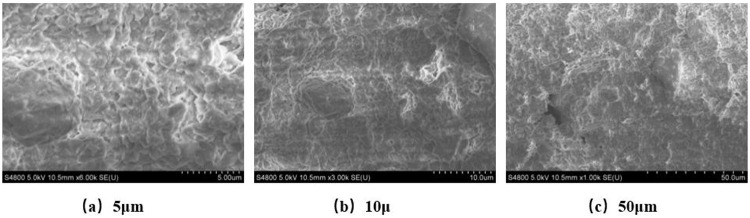
SEM analysis of sodium bentonite of friction reducing agent.

Fig 10 shows the microscopic structure images of sodium bentonite-based friction reducer under scanning electron microscopy. At 5μm scale, the friction reducer exhibits a layered stacking structure, with montmorillonite sheets arranged irregularly in flaky or book-like patterns, containing abundant nano-scale pores between layers, presenting as loosely stacked flaky structures, as shown in Fig 10(a). At 10μm scale, it shows a wide particle size distribution (approximately 1–10μm), with agglomeration phenomena observed in some areas, as shown in Fig 10(b). This morphology is directly related to high specific surface area and adsorption performance. At 50μm scale, the sodium bentonite after complete hydration forms a gel network, displaying interconnected pore networks, as shown in Fig 10(c).

#### 5.4.2. Friction reducer.

**Fig 11:**
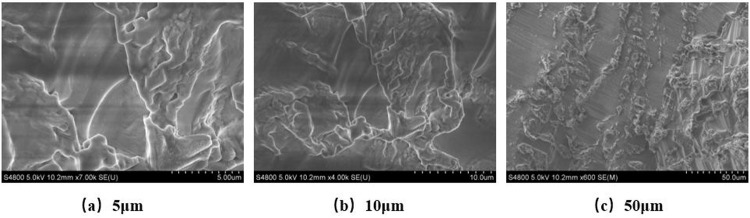
SEM analysis of SM – PAM - Na_2_CO_3_. (a) 5μm. (b) 10μm. (c) 50μm.

Fig 11 shows the microstructure of the graphite-polyacrylamide-sodium carbonate friction reducer under scanning electron microscopy. At 5μm scale, it shows obvious flake structure. Due to graphite’s strong conductivity, it appears brighter, as shown in Fig 11(a), which is a typical characteristic of graphite. At 10μm scale, it shows polyacrylamide molecules with fibrous network structure wrapping graphite and sodium carbonate, with no obvious agglomeration, forming a continuous phase, as shown in Fig 11(b). At 50μm scale, it shows relatively bright uniformly distributed flake graphite and darker polymer matrix. The synergistic effect of graphite, polyacrylamide and sodium carbonate forms a uniform and dense network structure, as shown in Fig 11(c). Graphite is uniformly dispersed and well-oriented, fully utilizing its interlayer sliding lubrication advantages to form an effective transfer film, significantly reducing friction and wear.

#### 5.4.3. Anti-permeability agent.

**Fig 12:**
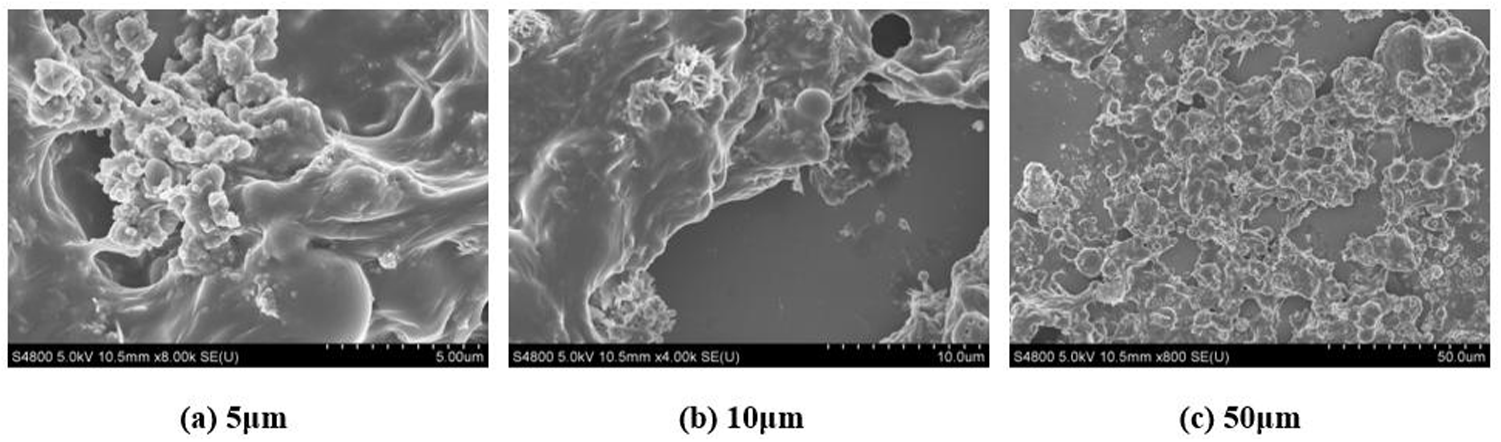
SEM analysis of polyurethane- Na_2_O·3.1SiO_2_. (a) 5μm. (b) 10μm. (c) 50μm.

Fig 12 displays the microscopic structure images of polyurethane- Na_2_O·3.1SiO_2_ under scanning electron microscopy. At the 5μm scale, it shows that the nano-SiO₂ particles generated from Na_2_O·3.1SiO_2_ decomposition fill the pores of the polyurethane polymer matrix, blocking the permeation channels. The polyurethane material forms a relatively continuous film on the substrate surface, initially creating a dense impermeability layer, as shown in Fig 12(a). At the 10μm scale, it shows well-dispersed Na_2_O·3.1SiO_2_ particles. The formed Si-O-C chemical bonds enhance the interfacial bonding strength, resulting in tight bonding between the Na_2_O·3.1SiO_2_ and polyurethane interface, as shown in Fig 12(b). At the 50μm scale, it shows that the polyurethane material formed by the reaction of isocyanate, polyol and other components presents a multilayer structure, as shown in Fig 12(c).

## 6. Formulation optimization

Based on orthogonal test results, a multi-index comprehensive evaluation system was established. By introducing the entropy weight method, the relative importance of fundamental performance indicators was quantified. Combined with multiple linear regression analysis, the nonlinear interaction relationships between factors and indicators were systematically analyzed to reveal the synergistic mechanisms of formulation component contents on the performance of friction reducers and anti-seepage agents.

### 6.1. Optimization of friction reducer formulation

#### 6.1.1. Comprehensive weight analysis of indicators.

**Fig 13:**
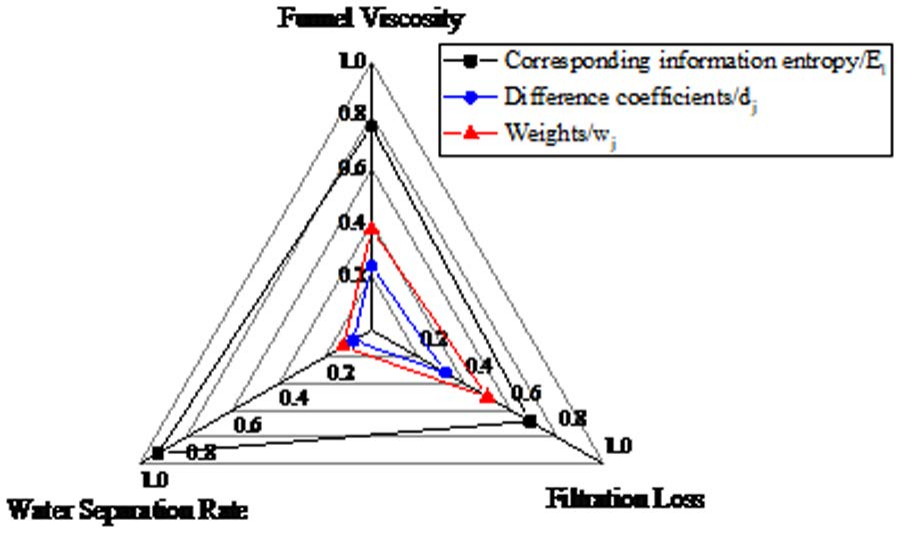
Radar chart of funnel viscosity, filtration loss and water separation rate.

The ranking by weight magnitude was: fluid loss (0.500)> funnel viscosity (0.376)> water separation rate (0.124). Radar charts were employed to characterize the relationships between fluid loss, funnel viscosity, water separation rate and their corresponding information entropy, difference coefficients, and weights, as shown in Fig 13.

#### 6.1.2. Interaction analysis between factors and indicators.

**Fig 14:**
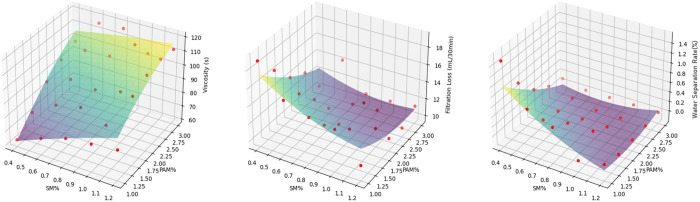
3D fitting of graphite, polyacrylamide and funnel viscosity, water loss and water separation rate.

Orthogonal experiments revealed that SM and PAM—the two most influential components in the friction reducer—exhibit identical or similar trends in their relationships with the indicators (funnel viscosity, fluid loss, and water separation rate). Python programming and the Linear Regression model were employed to fit three-dimensional surfaces depicting the relationships between these factors and indicators, as shown in Fig 14. Funnel viscosity, fluid loss, and water separation rate are primarily influenced by SM and PAM. For water-rich formations, appropriately increasing the content of SM and PAM can enhance funnel viscosity and reduce fluid loss, thereby effectively controlling water content and preventing instability in the friction reducer’s performance. For low-water-content formations, moderately reducing SM and PAM content ensures the friction reducer maintains effective friction reduction under such conditions. Adjusting the proportions of SM and PAM optimizes performance to meet the requirements of composite friction reducers in different geological conditions.

**Fig 15:**
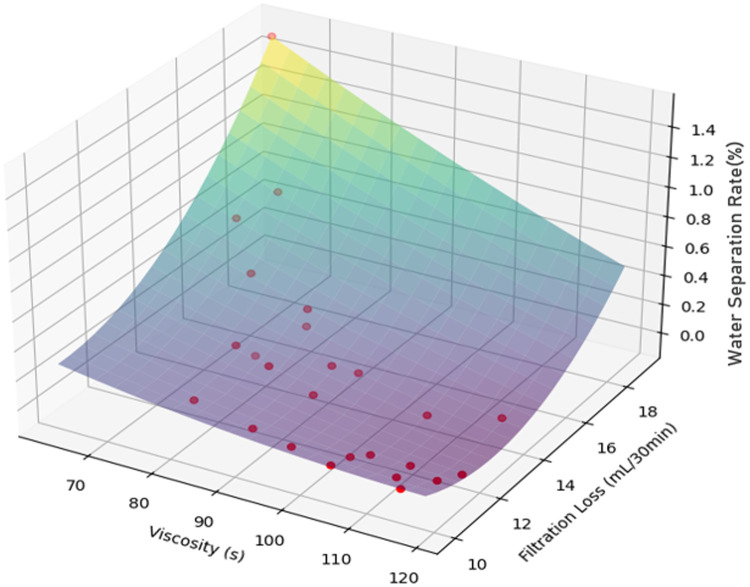
3D fitting of funnel viscosity, water loss and water separation rate.

Fig 15 demonstrates inherent correlations among funnel viscosity, fluid loss, and water separation rate: **Funnel viscosity vs. fluid loss**: An increase in funnel viscosity typically corresponds to a decrease in fluid loss, indicating that friction reducers with higher funnel viscosity better retain water and minimize leakage. **Funnel viscosity vs. water separation rate**: Higher funnel viscosity generally leads to lower water separation rates, suggesting improved water fixation and reduced water seepage. **Fluid loss vs. water separation rate**: Reduced fluid loss often coincides with a lower water separation rate, reflecting a positive correlation between the two. Minimizing fluid loss helps suppress water separation.

#### 6.1.3. Optimal friction reducer ratio.

Based on the preliminary analysis of the influence of various factors on performance indicators, the optimal composition range of the composite friction reducer is determined as follows: First, the optimal level of the core control indicator (fluid loss) is identified. As shown in Fig 8(b), the priority of factors affecting fluid loss is: Na₂CO₃ (C)> PAM (B)> SM (A). The optimal Na_2_CO_3_ (C) content for minimizing fluid loss is determined to be 0.6%. Next, the factor levels for the secondary important indicator (funnel viscosity) are adjusted. According to Fig 8(a), the influencing factors for funnel viscosity are prioritized as: PAM (B)> SM (A)> Na₂CO₃ (C). A moderate PAM dosage of 2.0%−2.5% is selected to increase viscosity without causing excessive thickening. Finally, the ratio for the minor indicator (water-separation rate) is optimized. Fig 8(c) shows that the influencing factors follow the order: PAM (B)> SM (A)> Na₂CO₃ (C). When the PAM content is maintained at 2.0%−2.5%, the water-separation rate is minimized, approaching 0, which is ideal. Since SM has a similar effect on water-separation rate as PAM, an optimal range of 0.8%−1.0% is selected. In summary, considering cost-effectiveness, the recommended composition ranges are: SM: 0.8%−1.0%; PAM: 2.0%−2.5%; Na₂CO₃: 0.3%−0.5%.

### 6.2. Optimization of anti-seepage agent ratio

#### 6.2.1. Analysis of comprehensive weighting indicators.

**Fig 16:**
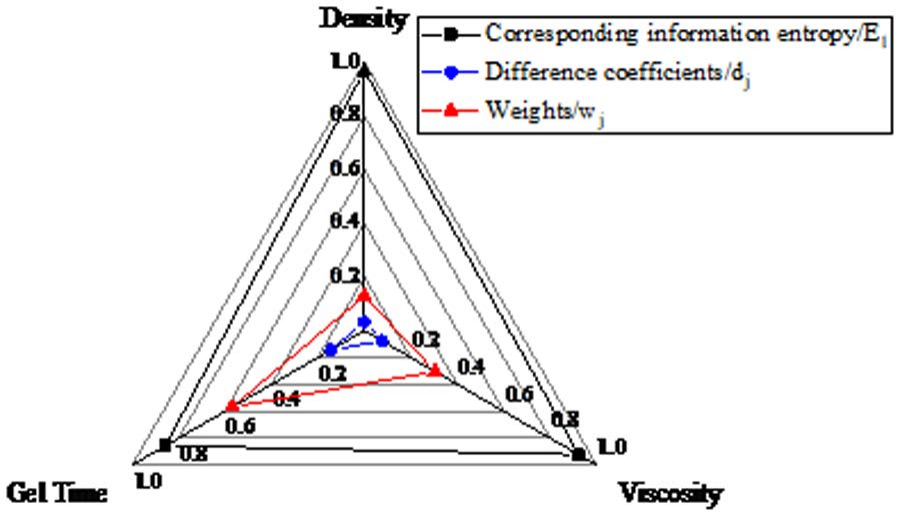
Radar chart of density, viscosity and gel time.

The ranking based on weight values is as follows: gel time (0.129)> viscosity (0.304)> density (0.568). A radar chart is used to illustrate the relationships among gel time, viscosity, density, and their corresponding information entropy, difference coefficients, and weights, as shown in Fig 16.

#### 6.2.2. Analysis of factor-indicator interactions.

**Fig 17:**
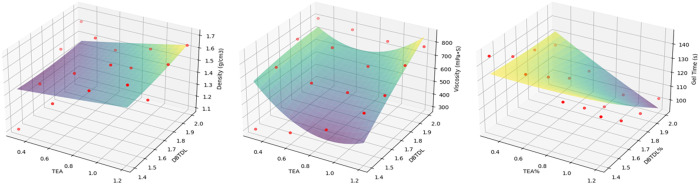
3D fitting of TEA, DBTDL with density, viscosity and gel time.

Based on the orthogonal experiment of the anti-seepage agent, it was found that TEA and DBTDL, the two most influential components in the anti-seepage agent, exhibit consistent or similar trends with respect to the indicators of density, viscosity, and gel time. Python programming and the LinearRegression model were employed to fit three-dimensional surfaces illustrating the relationship between factors and indicators, as shown in Fig 17. The synergistic effect of TEA and DBTDL significantly enhances the overall performance of the anti-seepage agent. Their combined action effectively increases density and viscosity while regulating gel time, thereby improving rheological properties and stability. In practical engineering applications, the performance can be optimized by adjusting the content of TEA and DBTDL. For scenarios requiring higher density and viscosity, the content of TEA and DBTDL can be appropriately increased, whereas for cases needing faster gel, the content of DBTDL can be reduced.

**Fig 18:**
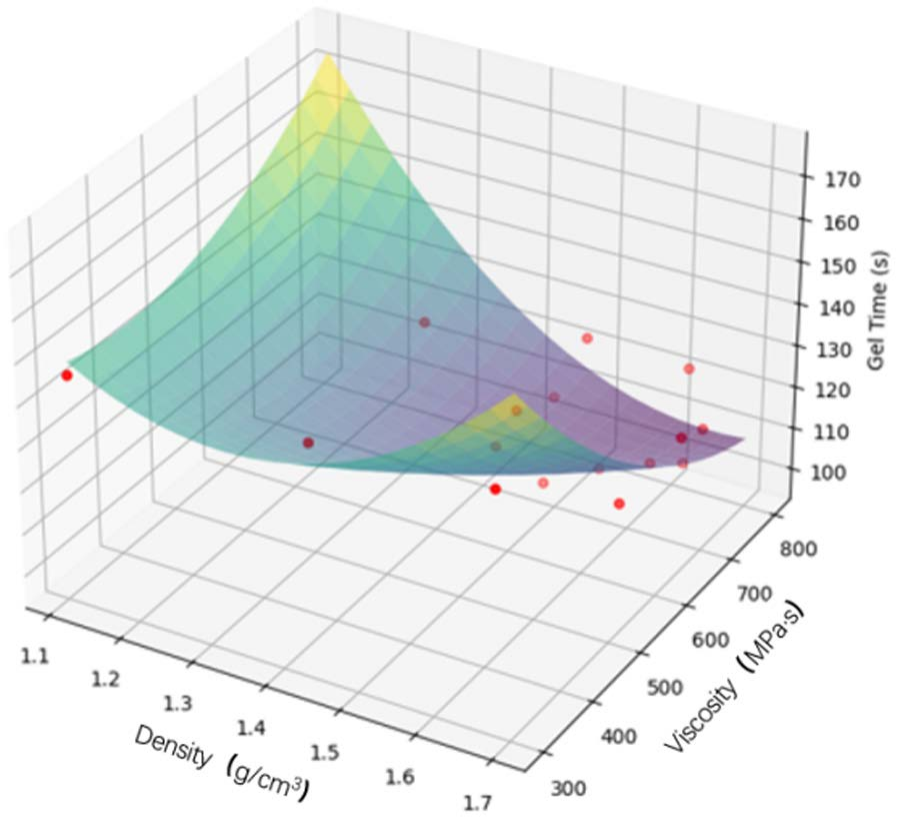
3D fitting of density, viscosity and gel time.

Fig 18 reveals certain intrinsic relationships among density, viscosity, and gel time: **Density and Viscosity**: An increase in density is typically accompanied by an increase in viscosity. This suggests that anti-seepage agents with higher density generally exhibit higher viscosity, which helps maintain structural stability. **Density and Gel Time**: An increase in density usually leads to an extended gel time. This indicates that anti-seepage agents with higher density require more time to form a gel, likely due to the complexity of their internal structure. **Viscosity and Gel Time**: An increase in viscosity is often associated with a longer gel time. This implies that anti-seepage agents with higher viscosity need more time to gel, possibly due to stronger intermolecular interactions within the material.

#### 6.2.3. Optimal anti-seepage agent formulation.

Based on the preliminary analysis of the influence priority of various factors on different performance indicators, a comprehensive evaluation was conducted to determine the optimal composition range of the anti-seepage agent.

First, the optimal factor level for the core control indicator (gel time) was determined. As shown in Fig 9(c), the priority of factors affecting fluid loss is: DBTDL (B)> TEA (A)> Na_2_O·3.1SiO_2_ (C). When the gel time is around 120 s (within the optimized range of 98–146 s), the dosage of B generally falls between 1.4% and 1.6%. This ensures sufficient penetration of the slurry into fractures while avoiding overly rapid solidification that could lead to incomplete filling. Next, the factor levels for the secondary important indicator (viscosity) were adjusted. Fig 9(b) shows that the priority of factors affecting viscosity is: DBTDL (B)> TEA (A)> Na_2_O·3.1SiO_2_ (C). B accelerates crosslinking, leading to increased viscosity, while a higher concentration of C also raises viscosity. When A is maintained at 1%, its steric hindrance effect slightly reduces viscosity. Finally, the formulation for the minor indicator (density) was optimized. As illustrated in Fig 9(a), the priority is: Na_2_O·3.1SiO_2_ (C)> TEA (A)> DBTDL (B). The density of Na_2_O·3.1SiO_2_ itself ranges from 1.3 to 1.5 g/cm^3^. When C ≥ 15%, the composite system’s density naturally exceeds 1.0 g/cm^3^, meeting the performance requirements.

In summary, considering cost-effectiveness, the recommended formulation ranges are: DBTDL (B): 1.4%−1.6%; TEA (A): 1.0%−1.5%; Na_2_O·3.1SiO_2_ (C): 15%−20%.

## 7. Conclusions

To address the incompatibility issue between bentonite-based friction reducers and anti-seepage agents, this study developed a composite anti-seepage friction reducer. This composite utilizes graphite for lubrication, Na_2_CO_3_ to maintain dispersibility, polyacrylamide to ensure thixotropy, and modified polyurethane to achieve anti-seepage performance. A grouting process for sealing ring inflation using the composite anti-seepage friction reducer in pipe jacking construction was proposed. Macro and micro performance tests of the composite were conducted, and the optimal mix ratio was determined using the range analysis method and entropy weight method. The main conclusions are as follows:

(1) The composite anti-seepage friction reducer outperforms traditional bentonite-based friction reducers in friction reduction, as evidenced by: a 55% reduction in funnel viscosity, a 25% decrease in filtration loss, a 4.5% reduction in water-separation rate (close to 0), and a 20%−28% decrease in friction coefficient. Orthogonal experiments revealed that polyacrylamide has the greatest influence on funnel viscosity, water-separation rate, and friction coefficient, while Na_2_CO_3_ most significantly affects filtration loss. The optimal friction reducer mix ratio ranges are: SM 0.8%−1.0%, PAM 2.0%−2.5%, and Na_2_CO_3_ 0.3%−0.5%.
(2) In terms of anti-seepage performance, the composite maintains a density above 1.0 g/cm^3^, a stable viscosity of 300–800 MPa·s, a gel time controlled within 98–146 s, and a permeability coefficient of the polyurethane anti-seepage agent mixed with standard sand meeting <10^-6^ cm/s, which satisfies the requirements for water-rich sand layers. Orthogonal experiments showed that Na_2_O·3.1SiO_2_ has the greatest influence on density, while DBTDL most significantly affects viscosity, gel time, and permeability coefficient. The optimal anti-seepage agent mix ratio ranges are: DBTDL 1.4%−1.6%, TEA 1.0%−1.5%, and Na_2_O·3.1SiO_2_ 15%−20%.

## Supporting information

S1 Fig Schematic model for friction coefficient test. (JPG)
