# Supplementary figures and images for: Preparation and performance of composite agent with anti-seepage and friction reducing for pipe jacking in water-rich sand stratum
Source: PLoS One. 2026 Feb 13;21(2):e0341338. doi: 10.1371/journal.pone.0341338 (PMC12904460; doi:10.1371/journal.pone.0341338)

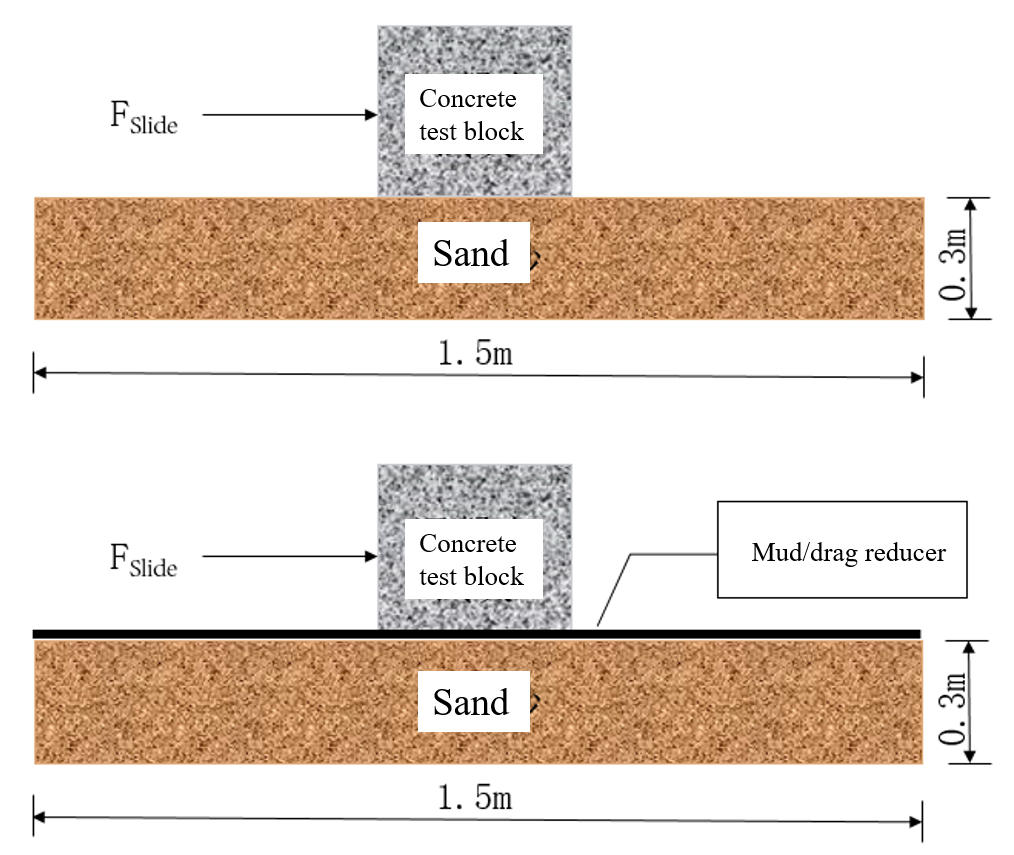

Supplement: S1 Fig — (JPG) [file pone.0341338.s001.jpg]
